# Exploring the 3D Printability of Engineered Cementitious Composites with Internal Curing for Resilient Construction in Arid Regions

**DOI:** 10.3390/ma18143327

**Published:** 2025-07-15

**Authors:** Tayyab Zafar, Muhammad Saeed Zafar, Maryam Hojati

**Affiliations:** Gerald May Department of Civil, Construction, & Environmental Engineering, University of New Mexico, Albuquerque, NM 87131, USA; tzafar@unm.edu (T.Z.); mszafar@unm.edu (M.S.Z.)

**Keywords:** 3D-printed concrete (3DPC), engineered cementitious composites (ECCs), internal curing, lightweight aggregates (LWAs), sustainability in construction, printability, pumice

## Abstract

This study investigates the feasibility of pumice-based internal curing based on the 3D printability of engineered cementitious composites (ECCs) for water-scarce environments and arid regions. Natural river sand was partially replaced with the presoaked pumice lightweight aggregates (LWAs) at two different levels, 30% and 60% by volume, and 50% of the cement was replaced with slag to enhance sustainability. Furthermore, 2% polyethylene (PE) fibers were used to improve the mechanical characteristics and 1% methylcellulose (MC) was used to increase the rheological stability. Pumice aggregates, presoaked for 24 h, were used as an internal curing agent to assess their effect on the printability. Three ECC mixes, CT-PE2-6-10 (control), P30-PE2-6-10 (30% pumice), and P60-PE2-6-10 (60% pumice), were printed using a 3D gantry printing system. A flow table and rheometer were used to evaluate the flowability and rheological properties. Extrudability was measured in terms of dimensional consistency and the coefficient of variation (CV%) to evaluate printability, whereas buildability was determined in terms of the maximum number of layers stacked before failure. All of the mixes met the extrudability criterion (CV < 5%), with P30-PE2-6-10 demonstrating superior printing quality and buildability, having 16 layers, which was comparable with the control mix that had 18 layers.

## 1. Introduction

Three-dimensional printing, also known as additive manufacturing or digital fabrication, has been extensively explored with cementitious materials in academia and industry over the last few years. The scale of projects varies from lab scale to the actual construction scale, for example, houses or prefabricated 3D-printed components for buildings or pedestrian bridges. The potential advantages associated with 3D printing technology include construction efficiency, geometric flexibility, sustainability, automation, and reduced project costs [1,2]. Although there have been many advancements in this technology, several challenges and research gaps still need to be addressed since several conventional approaches are not compatible with the 3D printing process. The key challenges include insufficient rheology and incompatibility with conventional reinforcing and curing techniques.

Three-dimensional concrete printing is a free-form construction technique that does not utilize any formwork like conventional construction practices. Although this factor contributes to construction cost reduction, due to the free-form nature of digital manufacturing and the layered structure, the printed structure is more prone to shrinkage cracks and void formation due to direct exposure to the environment, which results in the evaporation of the mixing water at a faster rate compared to the conventional construction methods [3]. Furthermore, this factor can also reduce the bond strength between the interface of the printed layers, as surface moisture conditions are crucial to prevent this [4]. Conclusively, negative pressure is induced on the pores by the evaporation of free water from the material, resulting in shrinkage cracking. Therefore, it is desirable to maintain the internal relative humidity to prevent moisture loss, promoting hydration reaction and improved interlayer bond strength [5]. The conventional external curing methods cannot transport significant amounts of moisture into cementitious materials with a lower water–binder ratio. Therefore, internal curing methods will come into play to provide water internally, avoiding self-desiccation. The most commonly used internal curing methods include porous materials such as lightweight aggregates (pumice, expanded shale, perlite, zeolite) [6,7,8,9], superfine powders (bottom ash), and physical water-absorbing materials (super absorbent polymers, bentonite clay) [2,7,8].

LWAs are porous materials and can be divided into three different types based on the source of the materials, such as natural LWAs (pumice), artificial LWAs (expanded perlite), and industrial wastes and recycled aggregates (expansion slag beads and cenospheres) [8]. LWAs, due to their porosity and internal pore structure, can be used as an internal curing agent. Meng et al. [10] found that saturated lightweight sand reduces autogenous shrinkage and enhances mechanical strength. Sedghi et al. [11] developed the 3D-printed lightweight cementitious mixtures by utilizing different grades of pumice and expanded glass. Moreover, the technical reports ACI (308-2-13) R-13 [12] and NISTIR 7765 [13] have summarized an in-depth review on the utilization of lightweight aggregates as an internal curing agent. Nevertheless, apart from the potential of LWAs as an effective internal curing agent, it is very crucial to use the optimal proportions, as high contents of LWAs in a cementitious mix can adversely degrade the mechanical performance [14,15,16].

A major challenge impeding the widespread acceptance of 3D concrete printing in civil infrastructure lies in the complexity of incorporating reinforcement into printed structural elements to ensure robust performance under various loading conditions. The conventional method of reinforcing materials is unsuitable for 3D-printed materials, necessitating essential material and reinforcement strategy modifications to make 3D printing a viable construction practice [17,18,19]. The commonly used reinforcing methods in 3D concrete printing include pre-installed, post-installed, and in-process reinforcement [20]. These reinforcement techniques include manually placed steel bars [21], a mesh mold [22,23], cables [24,25], nail/needle reinforcement [26,27], barbed wires [28], and prestressed reinforcement [29] However, most of these methods are not fully compatible with 3D printing processes as they can restrict nozzle movement during printing and they lack full automation for the entire construction process. In contrast, incorporating fiber reinforcement into the fresh concrete mixture during printing is deemed more compatible and practical for 3D printing technology. Previous studies have reported different types of fibers like metallic fibers, polyvinyl alcohol (PVA) fibers, glass fibers, carbon fibers, and basalt fibers used as concrete reinforcement [30]. Therefore, a self-reinforced ECC mixture with superior properties emerges as a novel candidate for 3D printing in construction, aiming to enhance the ductility (softening) and tensile capacity (hardening) of 3DPC elements through the in-process reinforcement technique [18]. However, adjustments in the fresh characteristics of the ECC mixture are necessary to meet the requirements for 3D printing.

This study is focused on utilizing saturated lightweight aggregates (pumice) as an internal curing agent in 3D-printed engineered cementitious composites (ECCs) to explore its effect on the printability of ECCs. The fibers used for ECCs include two different lengths of polyethylene (PE) fibers. The fresh properties, such as the flow table and rheology, were studied to gauge the effect of pumice on flowability and rheology. Furthermore, the printability of the 3D-printed ECC mixes was investigated in terms of extrudability and buildability.

## 2. Materials and Methods

### 2.1. Materials

The constituents of the mix include ordinary Portland cement (C), ground granulated blast-furnace slag (S), a high-range water-reducer agent (HRWRA), river sand (RS), lightweight aggregates, and PE fibers. C was provided by GCC Cement in Tijeras, NM, USA confirming the ASTM C150 standard [31] for type I/II cement. The chemical compositions of the binders (i.e., C and S) and pumice are shown in Table 1. In addition to the binder (B) components (C + S), methylcellulose (MC) was used as a rheology modifying agent and polyethylene (PE) fibers were added to provide acceptable ductility and tensile capacity [32]. The properties of these materials are reported in Table 2 and Table 3.

**Figure 1:**
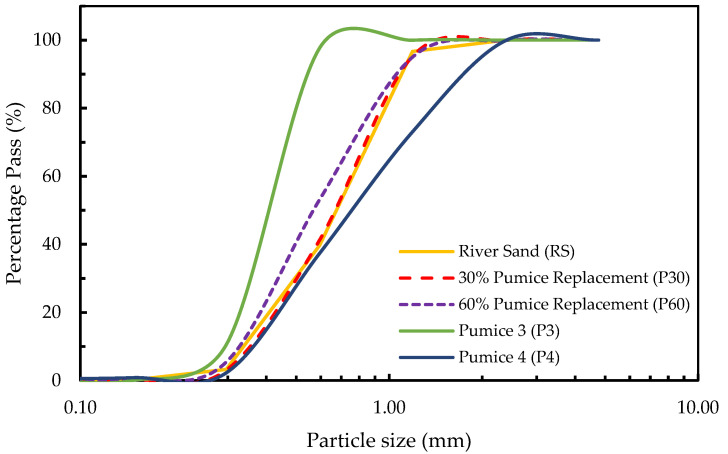
The gradation curves of the river sand (RS) and its blends with pumice.

**Figure 2:**
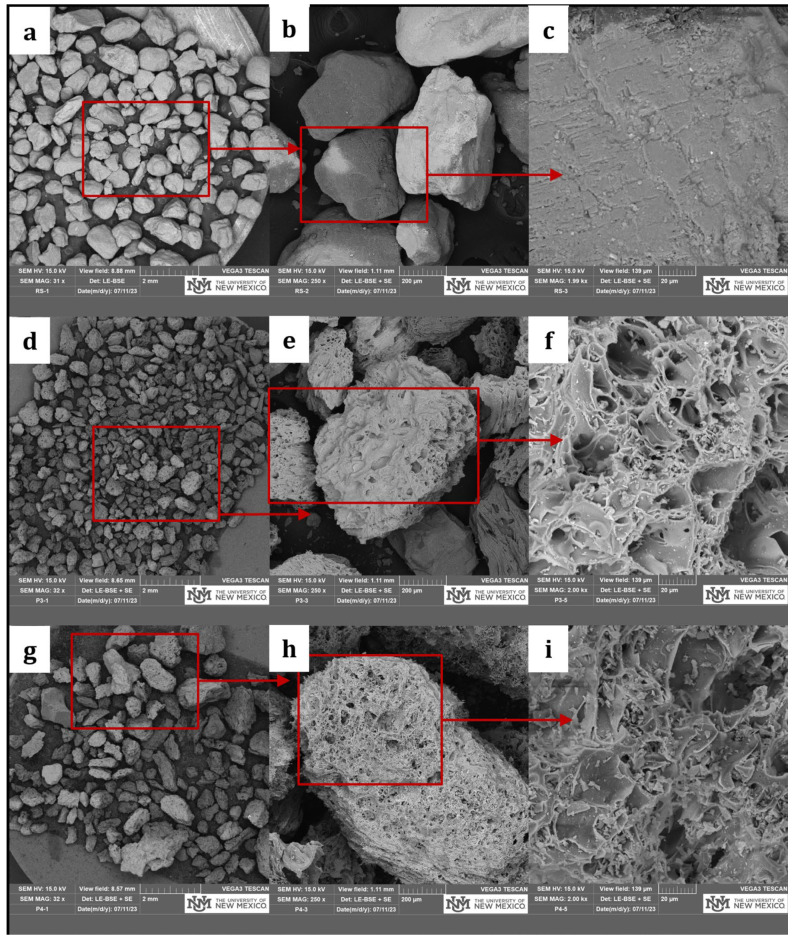
SEM micrographs of river sand (**a**–**c**), pumice 3 (**d**–**f**), and pumice 4 (**g**–**i**) at different magnifications.

Figure 1 illustrates the particle size distribution of river sand (RS), pumice (P3 and P4), and their blends with pumice at two replacement levels, 30% (P30) and 60% (P60). The gradation of the fine aggregates was carried out using the standard test method for the sieve analysis of fine aggregates conforming to ASTM C136/C136-19 [33]. Well-graded curves were observed for all of the fine aggregates, showing a continuous distribution of particle sizes ranging from 0.1 mm to 2 mm. With a fineness modulus (FM) of 2.60 and an average particle size (d_50_) of around 0.7 mm, RS showed a smooth and continuous gradation curve, representing uniform particle packing. P3, with an FM of 1.92 and an average particle size of 0.4 mm, displayed a uniformly graded curve. In contrast, P4 had an FM of 2.87 with a d_50_ of 0.8 mm, showing a well-graded curve with all particle sizes. Additionally, P30 had an FM of 2.60 with an average particle size of around 0.7 mm, and its gradation curve also overlapped the RS curve, representing a balanced particle size distribution conforming to a similar packing density and enhanced internal friction in the composite. In contrast, P60 had a fineness modulus of 2.46, with an average particle size of 0.6 mm, representing fine gradation. Scanning electron microscopy (SEM) was carried out using JEOL 5800LV SEM (JEOL, Tokyo, Japan) furnished with the secondary and backscattered electron and cathodoluminescence (CL) imaging detectors located at the Institute of Meteoritics, University of New Mexico. Scanning electron microscopy was performed to characterize the morphology of fine aggregates, as shown in Figure 2, which revealed distinct differences; RS particles were angular with a dense and smooth surface texture, resulting in stable particle interlocking. In contrast, pumice particles were highly porous with interconnected pores and a vesicular microstructure. Internal curing is facilitated by this porous morphology of pumice, which affects the rheology and hydration of composites.

### 2.2. Mix Design

A control mixture was created using RS as the fine aggregate, with an average particle size of d_50_ = 0.7 mm, specific gravity of 2.59, and water absorption capacity of 0.44%. A total of 50% of the mineral admixture S was replaced by the weight of cement in various mixtures. Two grades of pumice (pumice 3 (P3) and pumice 4 (P4)) were used as internal curing agents in the designed ECC for ambient-temperature environments with a specific gravity of 1.35 and water absorption capacity of 52%. The water absorption capacity of pumice was determined for 72 h using the paper towel method. Pumice was supplied by CR Minerals, which is located in Ohkay Owingeh, NM, USA. P3 and P4 were carefully blended to ensure an optimal particle size distribution. Internal curing was achieved by partially replacing the RS with a pre-wetted lightweight aggregate (pumice) to provide sufficient water for internal curing within the matrix during hydration to hydrate the hydrated cement particles to a maximum level. The mass of lightweight aggregates required for internal curing was calculated using the following equation [13]. According to calculations, about an 80% replacement of RS was required to fully hydrate the mix, but that would have badly impacted the mechanical performance; therefore, two pumice replacement levels were selected: 30% and 60%. Prior studies have also reported that optimal results are obtained by replacing between 10% and 30% of the LWA [34].

(1) $$M_{LWA}=\frac{C_{f}\times CS\times \alpha_{max}}{S\times \emptyset_{LWA}}$$

where

*M_LWA_* = mass of lightweight aggregates

*C_f_* = binder content

*CS* = chemical shrinkage

α*_max_* = Degree of hydration

*S* = Degree of saturation

∅*_LWA_*= Absorption

After finding the mass of LWA required for internal curing, the mass of water was calculated based on the water absorption capacity of the LWA and was expressed in the form of We/B for different levels of pumice replacements.

All mixtures included ADVA 195, and a high-range water reducer (HRWR) conforming to ASTM C494 [35], at a proportion of 0.0015% by weight of the binder content. Additionally, a viscosity-modifying admixture (MC), detailed in Table 3, was incorporated to alter the rheological properties and enhance the printing quality. PE fibers were added to each mix at a volume of 2% of the total mixture to mitigate early-age cracking in the printed filaments. The motivation behind using 2% volumetric fibers was due to the results of previous research [18,32] where the group investigated the comprehensive feasibility of ECC printability and mechanical performance by using different dosages of polyvinyl alcohol (PVA) and PE fibers, and the research group was successful in developing ultra-ductile ECCs with 11.9% strain capacity. Moreover, the rationale behind choosing 1% 6 mm fibers and 1% 10 mm fibers was to enhance the extrudability and tensile capacity, respectively. The mix design proportions for 3D-printed ECCs are outlined in Table 4. In the mix ID, taking P30-PE2-6-10 for instance, P30 represents a 30% pumice replacement with RS; PE2-6-10 stands for a 2% volume fraction of PE fibers with 1% each of 6 mm and 10 mm lengths.

### 2.3. Mixing Procedure

**Figure 3:**
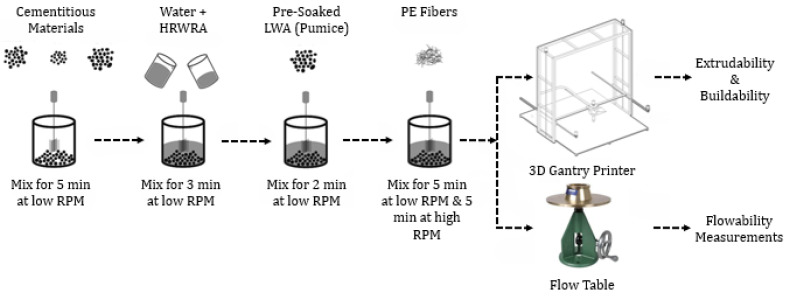
Schematic of the mixing and printing of ECCs.

The modified mixing procedure was adapted from [18] and further refined to enhance fiber dispersion and ensure uniform integration of presoaked lightweight pumice aggregates. Mixing was conducted in four stages as shown in Figure 3. First, the binders and fine aggregates were mixed at a low speed (140 ± 5 RPM) for 5 min. Next, a solution of water and HRWRA was added to the dry mix, followed by 3 min of additional mixing. Then, presoaked pumice aggregates were introduced into the wet mix. Finally, PE fibers were added, and mixing continued for 5 min at a low speed (140 ± 5 RPM), followed by 5 min at a high speed (285 ± 10 RPM).

### 2.4. Fresh Properties

#### 2.4.1. Flow Table Test

The flowability of the cementitious materials is the critical parameter that directly influences the printability of the designed mixtures. For improved extrudability, the designed mix is required to have high flowability, and after printing the filaments, it is preferred to have low flowability so that increased buildability can be achieved. Therefore, the flow table test is a good indicator to give sufficient information about the rheological characteristics of the designed mix. This test was performed according to ASTMC1437–20 [36] to adjust the water content in each mix. After mixing the material, the cone was placed at the center of the table and filled in two layers. Each layer was tamped 20 times, and then excessive material was removed from the top with the help of a spatula. Afterward, the cone was lifted gently, and the table dropped 25 times within 15 s. The spread diameter was measured at four different locations, and an average value was taken. The test was repeated three times for each mix, and an average of these three readings was taken as a final value.

#### 2.4.2. Rheology Test

**Figure 4:**
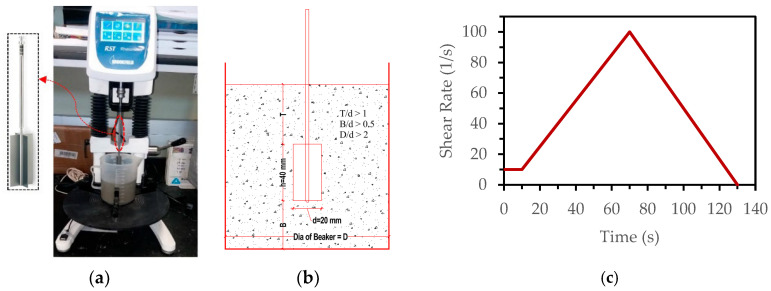
(**a**) Brookfield rheometer; (**b**) gap settings; (**c**) shear profile.

**Figure 5:**
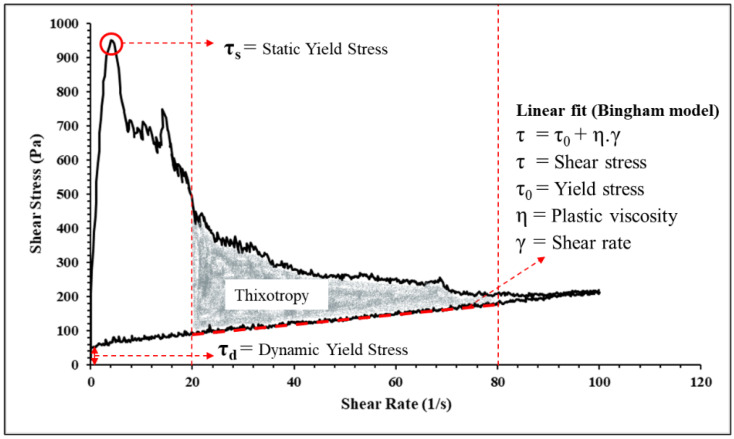
Bingham model illustrating various rheological parameters.

The rheological characteristics of the designed ECC mixtures were determined using the RST-SST Brookfield Rheometer. A VT-40-20 vane spindle was used, having a diameter of 20 mm and a length of 40 mm. From the literature [17], the hysteresis technique was used for measuring the rheological properties. Moreover, in order to avoid the boundary effects during the rheology test, the gap settings were ensured in line with those reported in the literature [37]. The test setup for the rheometer and the hysteresis loop is illustrated in Figure 4. Each of the designed ECC mix rheological measurements were made for a total duration of 130 s. For the first 10 s, the shear rate was kept at 10 s^−1^; then. for the next 60 s, the shear rate was increased from 10 s^−1^ to 100 s^−1^; then, for the last 60 s, it was decreased from 100 s^−1^ to 0 s^−1^. The Bingham model was used to identify the various rheological parameters, as shown in Figure 5. The measurements of the rheological parameters were taken in a controlled laboratory environment with regulated temperature and humidity. For each mix, three test repetitions were made, and an average value was used.

### 2.5. Three-Dimensional Printing System

**Figure 6:**
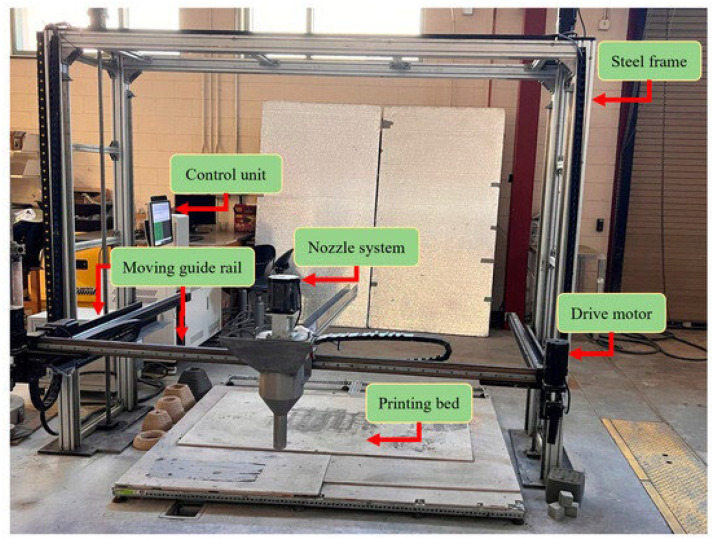
A 3D printer system and its integrated subsystems; “reproduced from Bhusal et al., 2023 licensed under CC BY 4.0 [38].

Figure 6 shows A gantry 3D printer housed in the Dana C. Wood Materials and Structures Lab at the University of New Mexico, which was used to evaluate the printability of the formulated mixes. This printer features a controller responsible for translating STL files into G-code and overseeing printing parameters such as the speed, extrusion rate, and layer height. With three linear degrees of freedom, the printer’s drive motors maneuver a 20 mm diameter circular nozzle along the X, Y, and Z axes. Print quality was evaluated based on the extrudability and buildability of the ECC mixes.

#### 2.5.1. Extrudability

**Figure 7:**
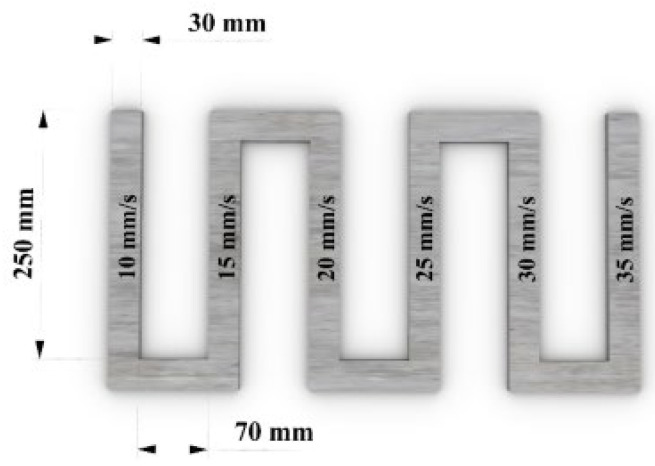
The extrudability test printing path for different printing speeds.

Extrudability is the ability of the material to pass through the nozzle and produce a continuous filament. It determines the quality and performance of the printed filaments in terms of the surface finish, uniformity, and dimensional accuracy. The extrudability of various mixtures was evaluated by printing a zigzag pattern with six sides, as shown in Figure 7. Each mixture underwent extrusion rate calibration at speeds of 0.1, 0.15, and 0.20 rounds per second to ensure material deposition. Printing speeds ranging from 10 mm/s to 35 mm/s were tested on each side of the pattern to determine the most favorable speed for consistent filament width. After printing, the filament width was measured to evaluate shape retention, uniformity, and reliability across mixtures and printing speeds. Finally, the six-sided path was again printed at the optimized speed and extrusion rate.

#### 2.5.2. Buildability

**Figure 8:**
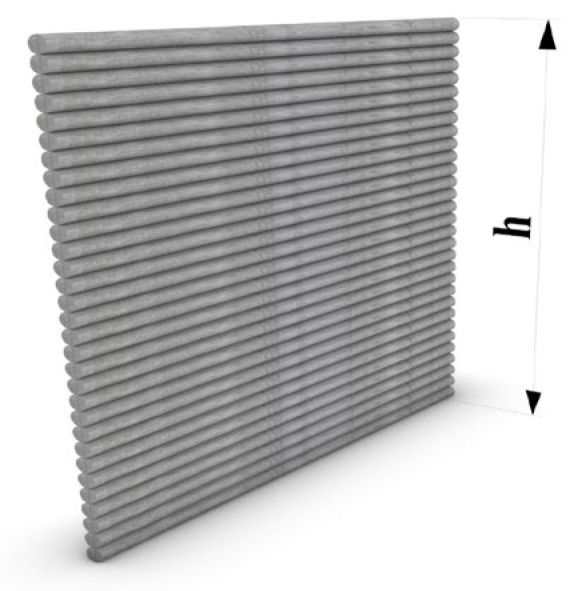
The buildability evaluation by 3D printing the wall.

Buildability is the material’s capability to withstand the printed filament’s self-weight and the weight of the subsequent layers without excessive deformations or buckling. Buildability was evaluated by printing a 20 mm × 400 mm wall with 20 layers, each layer having a 10 mm thickness, to measure the maximum height that each mixture can be printed to before failure occurs, as illustrated in Figure 8. This method was adopted from the literature [39] to assess the buildability of the designed ECC mixes.

## 3. Results and Discussion

### 3.1. Flowability

**Figure 9:**
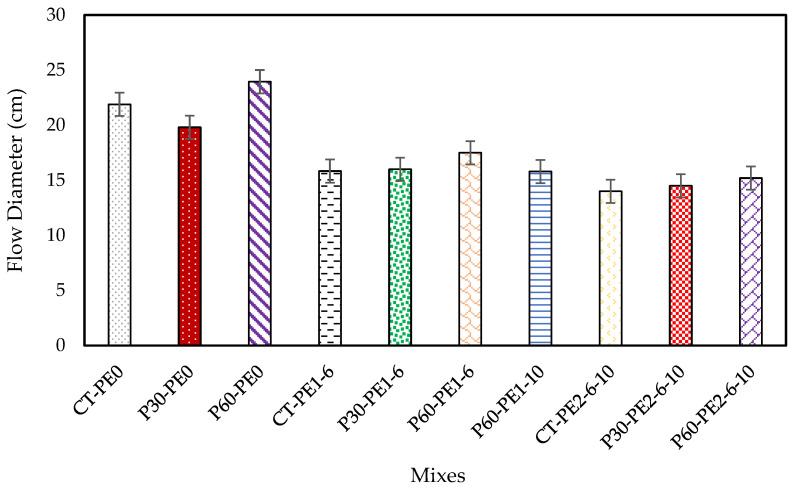
Flowability of plain and ECC mixes.

The rheological properties and extrusion performance of 3D-printed mixes depend on the mix’s flowability [40,41]. Figure 9 illustrates the flow table results for various plain and ECC mixes. Initially, ECC mixes were designed with a water-to-binder ratio of 0.27. Adjustments were made to this ratio to achieve flow diameters of between 12 and 15 cm for the ECC mixes (PE2), as previous research [18,42,43] has indicated that this range is ideal for producing flowable, pumpable, and buildable mixes before testing for printing. As C was replaced with S by up to 50%, and due to the higher water demand of S, the water-to-binder ratio was increased up to 0.31 so that ECC mixes with 2% PE fibers had a good enough flow that was within the above-mentioned range, ensuring printability. Additionally, the flowability of the plain mixes without PE fibers (PE0), as well as the ECC mixes with 1% PE fibers (PE1), was also determined to evaluate the effects of pumice replacement, fiber addition, and length of the fibers on the rheological properties of ECC mixes. MC was used as a viscosity-modifying agent in all of the mixes to enhance the printing quality; it significantly altered the flow behavior [18,39]. This could be attributed to the water-retention properties of cellulose ethers in ECC mixes, which prevent water migration between pores [44,45]. Additionally, the stickiness of the mix when in contact with the flow table surface can hinder its flow, and MC can cause the agglomeration of cement particles and hydration products, increasing dynamic yield stress and plastic viscosity, thus reducing flowability [46,47]. The flow diameter for ECC mixes with 2% PE fibers was in the range of 14 to 15 cm; the mixes appear to have good extrudability. Overall, a similar trend was observed across all of the mixes. To explore the effect of pumice replacement, a comparison was made between plain mixes without fibers, and a decrease of about 9.5% and an increase of 9.4% were observed in the spread diameters of P30-PE0 and P60-PE0, respectively. As saturated pumice aggregates were added to the mix to account for internal curing, they were expected to increase the flowability observed for P60-PE0. However, contrary to that, the decrease for P30-PE0 could be attributed to the aggregate shape effect. P4 and RS aggregates were angular and coarser in size than P3, which was granular with finer particles, but their replacement volume was low in this mix compared to the other mix: P60-PE0. A similar trend was recorded in the rheological parameters of P30-PE0, where an increase was observed in the static and dynamic yield stresses.

### 3.2. Rheology

In 3D concrete printing, it is essential to control the rheological parameters in order to produce a printable mix with adequate pumpability, extrudability, and buildability. Plastic viscosity, static yield stress, and dynamic yield stress are the rheological characteristics that must be carefully managed to produce enhanced quality printing [48,49]. Plastic viscosity is the ability of the fresh mix to resist deformations after exceeding the critical yield stress that occurs in the system. It is an important factor that controls the flowability and extrudability of mixes during printing. Static yield stress is the minimum shear stress required to initiate the flow; it is also referred to as the critical stress, the point at which the material deforms, and its structure begins to disintegrate. As it is associated with the material’s structure buildup, it is a key factor that plays an important role in buildability. After the structural breakdown occurs, the minimum stress that is required to maintain the material flow is referred to as dynamic shear stress [50]. It is related to the resistance to segregation, and it therefore plays an important role in pumpability and extrudability [18].

**Figure 10:**
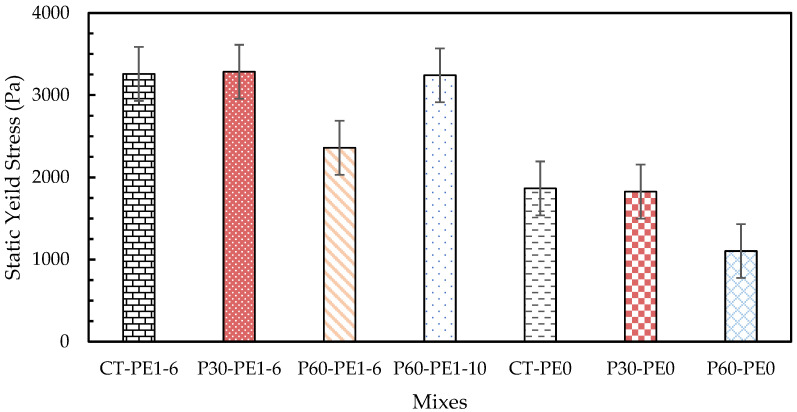
Static yield stress of plain and ECC mixes.

**Figure 11:**
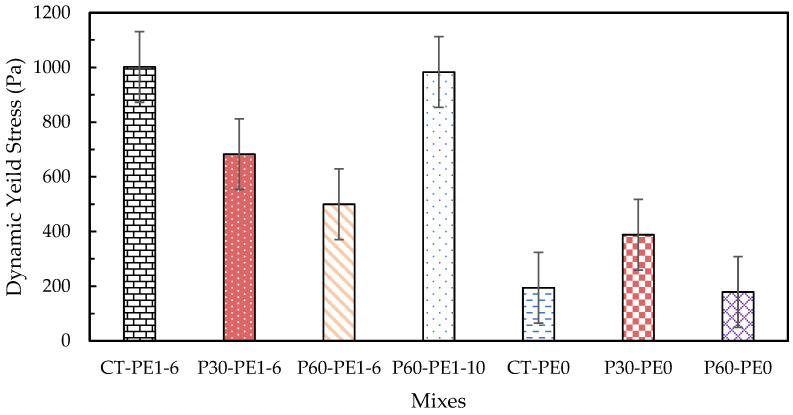
Dynamic yield stress of plain and ECC mixes.

**Figure 12:**
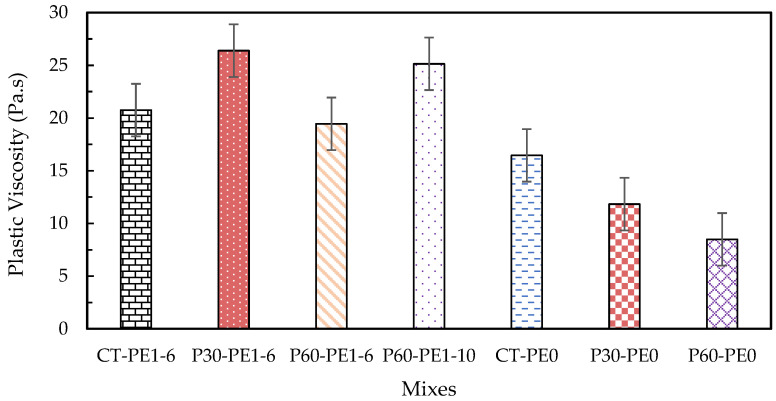
Plastic viscosity of plain and ECC mixes.

The rheology results of the plain and ECC mixes are illustrated in Figure 10, Figure 11 and Figure 12. At a 2% fiber dosage, the rheology tests failed due to the excessively high viscosity caused by the entanglement of long fibers. Therefore, tests were conducted using plain mixes without fibers as a baseline to assess the effect of pumice replacement, fiber addition, and fiber length on rheology. Additionally, the influence of fiber incorporation was studied separately by considering mixes with a 1% fiber dosage for both the 6 mm and 10 mm fiber lengths. Firstly, the effect of pumice replacement was observed by comparing the results of ECC mixes CT-PE1-6, P30-PE1-6, and P60-PE1-6 with 1% 6 mm PE fibers. The maximum static yield stress was observed for the control mix, whereas a 0.8% increase and a 27.6% decrease were observed for P30-PE1-6 and P60-PE1-6, respectively. The P30-PE1-6 mix showed an almost similar static yield stress as that of the control mix, indicating that the 30% pumice replacement did not significantly reduce the structural buildup, and the same was confirmed from the buildability of the P30 mix. However, P60-PE1-6 showed a significant reduction in the static yield stress, which can be attributed to the higher water content, increased porosity, and lower density of the pumice as compared to RS, which may have reduced the interparticle friction and resulted in enhanced flowability. Also, at 60% replacement, there were more fine particles that may have released the water at a faster rate resulting in this reduction, which aligns with the results of prior studies [34]. A 31.8% and 50.1% decline was observed in the dynamic yield stress of P30-PE1-6 and P60-PE1-6, respectively. In comparison, a slightly different pattern was noticed for the plastic viscosity of the mixes. P30-PE1-6 exhibited the highest viscosity, as compared to the other mixes. An increase of 27.2% was observed, whereas a 6.3% decrease was recorded for P60-PE1-6. This increase in viscosity can be attributed to the higher content of angular and coarser sized RS and pumice 4, which might have increased the degree of interlocking between the particles, resulting in a higher plastic viscosity.

Secondly, in order to evaluate the effect of fiber addition on the rheological parameters, a comparison between the fiber-reinforced mixes CT-PE1-6, P30-PE1-6, and P60-PE1-6 and their corresponding plain mixes CT-PE0, P30-PE0, and P60-PE0 was made. The introduction of PE fibers significantly increased the rheological characteristics of the mixes. An increase of 74.8%, 79.9%, and 114% was noted for the static yield stress, whereas an increase of 415.8%, 75.9%, and 179.5% was recorded for the dynamic yield stress of the mixes. A similar trend was observed for the plastic viscosity of the mixes. This increase is attributed to the formation of the internal structure due to the needle-shaped particles of the fibers [11,51]. Lastly, the effect of fiber length was evaluated by comparing the results for P60-PE1-6 and P60-PE1-10. An increase of 37.4%, 96.8%, and 29.3% in the static yield stress, dynamic yield stress, and plastic viscosity was observed. These results clearly demonstrate that by increasing the length of the fibers, fiber entanglement in the matrix is increased, which results in higher rheological properties; however, there is an increased risk of nozzle blockage with longer fibers, which also results in discontinuous 3D-printed filaments.

### 3.3. Extrudability

Extrudability is the crucial parameter for 3D-printable cementitious materials, which directly affects the printed filament uniformity, stability, and quality. The mixture design composition, including water, primarily controls the extrudability binder ratio, admixtures, aggregate content and properties, environmental conditions, rheological properties, and printing parameters like the printing and extrusion speed, nozzle shape, and nozzle offset from the printed surface [52].

**Figure 13:**
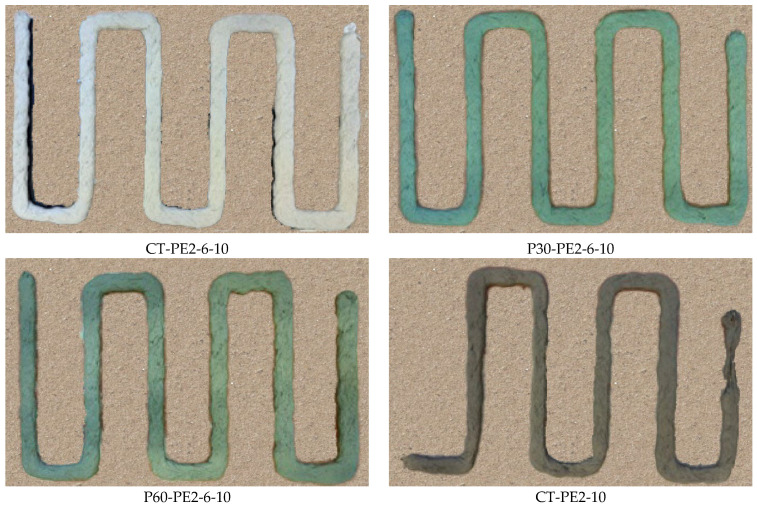
Extrudability of ECC mixes CT-PE2-6-10, P30-PE2-6-10, P60-PE2-6-10, and CT-PE2-10.

A criterion has been defined based on the standard deviation values to evaluate the extrudability of 3D-printed concrete filaments using the dimensional consistency of the printed elements. The coefficient of variation (CV) was calculated by dividing the standard deviation by the mean and expressed as a percentage. It offers an appropriate measure for dimensional uniformity, where lower CV values indicate a more stable extrusion process and higher consistency. In this study, a CV value of less than 5% is considered to show good dimensional consistency [18]. All of the ECC mixes shown in Figure 13 were examined using this criterion, and the mixes whose CV values lie in this specified range are deemed printable mixes.

**Figure 14:**
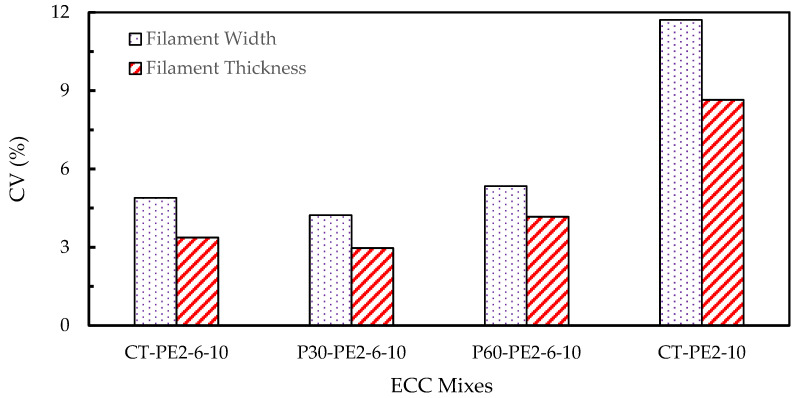
Extrudability of ECC mixes based on the coefficient of variation of filament width and thickness.

As is displayed in Figure 14, the lower CV values were observed for the mixes CT-PE2-6-10, P30-PE2-6-10, and P60-PE2-6-10 for both the filament width and thickness, which is an indication of good dimensional consistency. In contrast, CT-PE2-10 demonstrated higher variability in width (12%) and thickness (9%), indicating instability in the printed filament. This poor extrudability of CT-PE2-10 can be attributed to the high content (2%) of long fibers (10 mm), which are more likely to cause interlocking and entanglement, leading to pulsating extrusion flow and discontinuities in the printed filament. When the effect of the length of fiber on rheological properties was explored for P60-PE1-10 in comparison with P60-PE1-6, an increase in the dynamic yield stress and plastic viscosity was observed, which supports the above observation for CT-PE2-10, since excessive rheological resistance restricts the continuous flow and negatively impacts the shape of the filament.

In contrast, moderate rheological properties were observed for the control and modified mixes, namely, CT-PE2-6-10, P30-PE2-6-10, and P60-PE2-6-10. The dynamic yield stress and viscosity were in the range of 500–1000 Pa and 20–25 Pa.s, respectively. These rheological properties are suitable for 3D printing, as they offer enough cohesion to hold the shape of the filament without causing nozzle blockage. Adding short fibers (6 mm) improved the fiber dispersion and reduced the fiber entanglement, facilitating the extrusion process, whereas long fibers offered reinforcement benefits.

Moreover, the partial replacement of RS with presoaked lightweight aggregates (pumice) in mixes P30-PE2-6-10 and P60-PE2-6-10 improved the extrudability by reducing the internal friction and density, and improved the particle packing, as is evident from the slightly reduced dynamic yield stress values compared to those of CT-PE2-6-10. This flow behavior adjustment likely resulted in a smooth extrusion path and improved filament conformity with uniform and well-defined layers, as depicted in Figure 13. Meanwhile, for the CT-PE2-10 mix, wavy and irregular filaments were observed, which might be due to the large fiber entanglement. In conclusion, extrudability is strongly associated with rheological properties, specifically plastic viscosity and dynamic yield stress. Therefore, extrudability can be improved by optimizing the fiber length and dosage with presoaked pumice LWAs and keeping the rheological characteristics in a workable range.

### 3.4. Buildability

**Figure 15:**
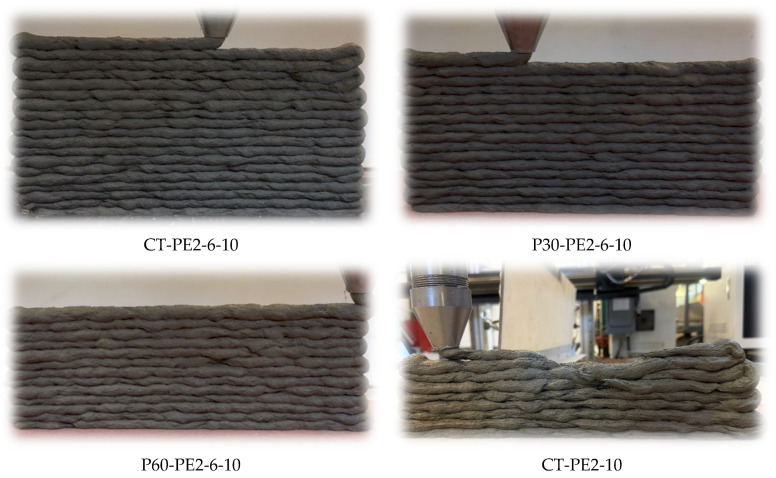
Buildability evaluation of ECC mixes CT-PE2-6-10, P30-PE2-6-10, P60-PE2-6-10, and CT-PE2-10.

In 3D concrete printing, buildability, also known as structural buildup, refers to the ability of the printed filaments or layers to withstand their self-weight and the weight of the subsequent layers without any failure in the fresh state [53,54]. This failure can be due to the deformation in the bottom layer, or it can be a buckling failure, depending upon the location of the failure. It can also be because of the self-weight of the layer, the weight of the following layers, and the extrusion pressure of the nozzle. Shape stability and buildability are the key objectives and play a vital role in evaluating design mixes for printability. Generally, shape stability and buildability are closely associated with the rheology of the mix, particularly the static yield stress, which plays an important role in structural buildup [55]. A 20 mm × 400 mm wall was printed to evaluate the buildability. Before printing, the wall extrudability of the mix was checked on the zigzag pattern, and optimum printing parameters were determined. The buildability evaluation of mixes CT-PE2-6-10, P30-PE2-6-10, P60-PE2-6-10, and CT-PE2-10 is presented in Figure 15 and Table 5 based on the maximum number of layers printed before the structural failure. For CT-PE2-6-10, the printing speed and extrusion rate were kept constant at 10 mm/sec and 0.10 rounds/sec, and a maximum of 18 layers were printed before failure. For P30-PE2-6-10 and P60-PE2-6-10, the printing speed was 10 mm/sec, the extrusion rate was kept constant at 0.10 rounds/sec, and the maximum number of layers printed was 16 and 13, respectively. In contrast, only five layers were printed for CT-PE2-10 before failure.

This variation in the buildability of ECC mixes is well aligned with the static yield stress values of the mixes illustrated in Figure 10. High static yield stress values were observed for the CT-PE2-6-10, P30-PE2-6-10, and CT-PE2-10 mixes, which is beneficial for upholding the structural integrity of the printed layers. Static yield stress is the minimum stress at which a material transitions from a solid-like state to a flowable state. A higher static yield stress indicates that the printed filament will resist the deformation and weight of the following layers after extrusion.

Despite CT-PE2-10 having a higher static yield stress, its buildability was restricted to only five layers, which is an indication that static yield stress alone is not sufficient, and rheological instabilities introduced by the high fiber content and length of fibers are the factors that are likely to cause poor interlayer bonding or structural instability at greater heights, underscoring the importance of the rheological fiber interaction.

On the other hand, the lowest buildability (13 layers) was observed for P60-PE2-6-10 among the hybrid fiber mixes, correlating with its lower static yield stress. This mix contained a high volume of presoaked pumice LWAs, which might have caused a reduction in the static yield stress and buildability due to an increased moisture content at an early age limiting the vertical buildup. It can be inferred that buildability positively correlates with the static yield stress, with higher values supporting better shape retention and layer stacking. However, controlled fiber architecture and particle gradation combined with a high static yield stress result in optimal buildability, facilitating the mix to resist failure while sustaining extrudability.

## 4. Conclusions

The aim of this study was to investigate the influence of internal curing using pumice LWAs as an internal curing agent on the 3D printability of engineered cementitious composites (ECCs), particularly modified for arid regions. Based on flowability, rheology, and printability evaluations, the following conclusions can be drawn:

1. The inclusion of presoaked pumice LWAs significantly affected the flowability of ECC mixes. Due to increased internal moisture availability, improved flowability was observed for the mix P60-PE2-6-10 with a higher replacement level (60%), whereas the mix P30-PE2-6-10 with a lower replacement level showed a reduction in flowability, which could be attributed to the coarser texture and particle angularity resulting in increased inter-particle friction.
2. The rheological characteristics of ECC mixes are substantially affected by the addition of presoaked pumice LWAs. Due to angular particle interlocking, the P30-PE2-6-10 mix sustained a similar static yield stress and increased plastic viscosity, whereas a reduction in static and dynamic yield stress was observed for mix P60-PE2-6-10, resulting in increased flowability due to enhanced internal moisture and porosity.
3. Moreover, adding long fibers improved the rheological properties, but the possibility of nozzle blockage was also enhanced due to fiber entanglement, which would likely restrict the continuous extrusion.
4. The extrudability test was performed to evaluate the printing quality of the ECC mixes with and without lightweight aggregates. All of the ECC mixes showed good extrudability except for CT-PE2-10, with a CV value of less than 5%; however, superior quality was observed for P30-PE2-6-10, maintaining the acceptable dimensional accuracy, underscoring the effectiveness of internal curing with sustained printing consistency.
5. The optimum printing quality was observed at a printing speed of 1 cm/s with an extrusion rate of 0.1 rounds/s, conforming to a smooth filament texture, dimensional consistency, and stable extrusion.
6. The addition of presoaked pumice LWAs caused a reduction in the buildability of ECC mixes, with a maximum of 18 layers for the control mix CT-PE2-6-10 as compared to 16 and 13 layers for P30-PE2-6-10 and P60-PE2-6-10, respectively. The modified mixes contained presoaked pumice lightweight aggregates, which released water with the passage of time and increased the water content, influencing the buildability.
7. Conclusively, it can be inferred that P30-PE2-6-10 demonstrated adequate shape stability and acceptable printability performance, indicating that moderate pumice replacement can balance the benefits of internal curing without impacting the structural integrity during printing.

## 5. Future Work

The next phase of this study will focus on evaluating the influence of internal curing on the mechanical performance of 3D-printed ECC mixes. Specifically, specimens will be cured under three different regimes: (1) moist curing, (2) sealed-chamber curing using saturated salt solutions, and (3) ambient lab conditions with variable humidity levels. These curing environments will help to assess the effect of moisture availability on compressive strength, flexural strength, and direct tensile strength. Microstructural characterization and thermogravimetric analysis (TGA) will be conducted to further understand the internal curing mechanism. Additionally, isothermal calorimetry will be employed to study the heat of hydration and its relation to internal curing dynamics.

Building upon the valuable feedback received, future research will also incorporate a broader sustainability and environmental performance evaluation. This includes quantifying potential water savings from internal curing during the mixing and post-printing phases and assessing evaporative losses under different curing conditions. A life-cycle perspective will be adopted to explore trade-offs between the use of pumice-based aggregates and conventional or locally available materials, considering factors such as transportation energy and environmental impact. Furthermore, the potential geochemical risks associated with pumice extraction, including heavy-metal content or leachability, will be examined based on the available datasets and environmental standards.

These extensions will provide a more holistic understanding of the printability–performance–environment nexus, contributing to the development of resilient, sustainable, and regionally adaptable cementitious systems for 3D printing in arid environments.

## Figures and Tables

**Table 1:** Chemical compositions of binders (C, S) and lightweight aggregates.

Material	SiO_2_	Al_2_O_3_	Fe_2_O_3_	CaO	MgO	SO_3_	K_2_O	TiO_2_	Na_2_O	Specific Gravity
C	19.24	4.75	3.35	65.80	2.20	3.61	0.54	0.21	-	3.13
S	30.80	11.45	2.26	47.50	3.65	3.03	0.38	-	0.17	2.91
Pumice	75.1	12.5	2.00	0.425	0.072	0.018	5.67	0.086	3.55	1.35

**Table 2:** Properties of PE fibers.

Material	Diameter (Microns)	Length (mm)	Specific Gravity	Tenacity (GPa)	Elastic Modulus (GPa)	Color
PE Fibers	17.9	6–10	0.97	4.0	114	White

**Table 3:** Properties of MC.

Material	Viscosity (cP)	Degree of Substitution	Methoxy Substitution (%)	Molecular Weight (g/mol)
MC	15	1.5–1.9	27.5–31.5	14,000

**Table 4:** Mix proportions of ECC mixes (weight-to-binder ratio).

Mix ID	C/B	S/B	RS	Pumice	W/B	We/B	Agg/B	MC (%) ^1^	HRWR (%) ^1^	PE Fibers (Vol%) ^2^
CT-PE2-6-10	0.5	0.5	1.0	0.0	0.31	-	0.3125	0.01	0.0015	2
P30-PE2-6-10	0.5	0.5	0.7	0.3	0.30	0.0206	0.3125	0.01	0.0015	2
P60-PE2-6-10	0.5	0.5	0.4	0.6	0.30	0.0411	0.3125	0.01	0.0015	2

Note: ^1^ HRWR and MC (% weight of the binder B = (C + S)); ^2^ 1% 6 mm and 1% 10 mm PE fibers (% of the total mix volume); We/B = ratio of internal curing water to the binder.

**Table 5:** Printability evaluation of the different ECC mixes.

Mix ID	Printing Speed (mm/s)	Extrusion Speed (RPS)	No. of Layers Printed	Buildable	Extrudable
CT-PE2-6-10	10	0.1	18	✓	✓
P30-PE2-6-10	10	0.1	16	✓	✓
P60-PE2-6-10	10	0.1	13	✓	✓
CT-PE2-10	10	0.2	5	✓	✕

## Data Availability

The original contributions presented in this study are included in the article. Further inquiries can be directed to the corresponding author.
